# Resident Physician Perceptions of Wellness Interventions: A Single-Institution Qualitative Study

**DOI:** 10.7759/cureus.104931

**Published:** 2026-03-09

**Authors:** June Criscione, Kiersten Flodman, Benjamin Doolittle

**Affiliations:** 1 Internal Medicine - Pediatrics, Yale School of Medicine, New Haven, USA; 2 Pediatrics, Yale School of Medicine, New Haven, USA

**Keywords:** health wellness and promotion, medical trainee wellness, residency wellness, wellness programs, workplace wellness

## Abstract

Introduction

Resident physicians are at high risk for burnout and mental health conditions, which negatively impact patient care. In response, many residency programs have implemented wellness initiatives. However, more research is needed on how residents themselves perceive specific interventions. Thus, we qualitatively assessed residents’ perceptions of graduate medical education (GME) administration-sponsored wellness interventions at an academic medical center.

Methods

Qualitative questions were adapted from a study on perceptions of workplace wellness initiatives and distributed to residents in May-June of 2024. An inductive thematic approach was used to identify common themes across aggregate responses.

Results

Seventy-eight respondents represented 18 residency programs. A slight majority of respondents identified as female (40/78, 51.3%). The largest proportion of respondents identified as White participants (36/78, 46.2%). Preferred wellness interventions included food, personal resources, and opportunities for social connections. Respondents had mixed views on giveaways. Respondents identified work hours and time off as major structural determinants of well-being. Respondents also referenced aspects of their work environments, including the negative impact of mistreatment and the importance of mentorship. Wellness initiatives that appear to favor certain residents, or that have limited availability for all residents, may negatively impact well-being.

Conclusions

Inclusion of residents from multiple programs provided broad input on GME-sponsored wellness initiatives. While social connections, food, and other resources enhance well-being, work environments and perceptions of inequity in wellness initiatives may negatively impact resident well-being. These themes may correspond with existing theoretical frameworks on physician well-being. Future multi-institutional studies would increase the reliability of these findings and inform improvements in GME-sponsored wellness initiatives.

## Introduction

Resident physicians are at high risk for burnout and mental health conditions, including depression and anxiety [1-3]. Although estimates vary across different assessments, some studies suggest that over 50% of residents experience symptoms of burnout, and 20%-35% of residents may suffer from depression, with as many as one in 16 residents in certain training programs experiencing suicidal ideation [1,2]. In addition, burnout and mental health conditions among residents may negatively impact patient care [1,2]. For example, one study found that residents screening positive for depression were more than six times as likely to make a medication error [4]. In response to such findings, many graduate medical education (GME) administrations and individual residency programs have implemented initiatives intended to improve resident well-being and mental health [1-3]. Although some studies have shown positive outcomes from specific interventions, there remains a paucity of evidence on how to best support resident well-being [1-3].

Preliminary data suggest that wellness initiatives must be multifaceted [3]. Interventions focused on individual residents, such as resilience or mindfulness training, are unlikely to have durable, positive impacts unless programs also address structural drivers of resident well-being, such as documentation burden and access to physical or mental health care [3]. Resident well-being may also be impacted by cultural norms that do not prioritize engaging in self-care [3]. Thus, residents must be informed about wellness interventions and feel comfortable accessing them [2]. Furthermore, research suggests that wellness initiatives are not one-size-fits-all. An intervention that helps one resident may not be beneficial to other residents [5]. Even common assumptions about beneficial interventions, such as work-hour restrictions, may not apply to all residents and may have unexpected negative effects on well-being [1]. One study, for example, showed no significant improvement in quality-of-life measures after work-hour reduction among surgical residents, a finding that may have been mediated by reduced patient continuity and lack of improvement in learning climate [6].

Involving residents in the design of wellness initiatives is a promising strategy for selecting effective interventions. Research suggests that employers and employees in medical care settings may have differing perceptions of workplace wellness opportunities [7]. Accordingly, studies have found that wellness initiatives driven by residents, who are most familiar with the factors impacting their own well-being, can have positive impacts on their perception of well-being, burnout, and work-life balance, among other measures [1,8]. Successful wellness initiatives also tend to solicit resident feedback [9]. However, there is limited information on how residents themselves perceive specific wellness interventions, and what these perceptions might demonstrate about broader mediators of resident or clinician well-being. A 2024 qualitative study by Warburton-Silva et al. successfully identified themes for resident-preferred wellness interventions, such as developing a supportive community, addressing basic needs, and acknowledging structural limitations. However, interpretation of these findings was limited by a small sample, comprised of a single residency program [10].

Building upon these limited findings, the primary aim of this study was to qualitatively assess how residents from multiple programs perceived wellness interventions carried out by the GME administration and a GME-sponsored, house staff-led wellness council at a large academic medical center. As a secondary aim, we also sought to identify general perceptions of factors impacting well-being, all with the goal of improving GME-sponsored wellness programming and providing insight into mediators of resident well-being.

Data in this article were previously presented as a meeting abstract at Yale School of Medicine Medical Education Day on June 6th, 2025.

## Materials and methods

Study design

Recognizing that variations in clinical schedules might favor focus group participation from residents in certain programs, this study centered instead on the use of an electronic survey, with several open-ended qualitative questions designed to capture a wide range of perceptions regarding wellness initiatives and resident well-being in general. The questions were unique to this study in both wording and topics addressed, but followed a similar structure to those included in a focus group guide in a 2019 study by Seward et al. on general employees' perceptions of workplace wellness opportunities at a large academic medical center - a federally funded study designated as HHS Public Access [7].

These questions were then compiled into an electronic survey using a secure online platform available through the academic medical center. The survey also included basic demographic questions, including residency year and program, approximate age, and racial and gender identity (see Appendix A for full survey). 

Informed consent and IRB approval

Informed consent was requested at the beginning of the electronic survey, and the study was granted an exemption through Yale University’s institutional review board (IRB# 2000037258). The GME office also approved this study and distributed the survey through institutional e-mail list-serves.

Study setting and participants

The electronic survey was distributed through a house staff e-mail list at Yale New Haven Hospital, a large academic medical center in New Haven, CT, USA, in the spring of 2024. The survey remained open from mid-March to early June, and house staff received several reminder notifications requesting their participation. Trainees in non-integrated fellowship programs were excluded from the study based on an assumption that different clinical responsibilities may lead to differences in well-being needs between resident and fellow physicians, leaving approximately 1,025 eligible trainees. Members of the wellness council were also excluded based on their involvement in carrying out GME-sponsored wellness interventions.

Data analysis

This study utilized a phenomenological approach to thematically analyze resident perceptions of wellness interventions and other factors impacting resident well-being [11,12]. Analyses were informed by a constructivist paradigm, which assumes a subjective reality based on individual experiences. In the context of this study, resident physicians were assumed to have constructed their own understanding of factors impacting well-being, based on their individual experiences [11,12].

Thematic analysis was performed using inductive coding to identify common themes across aggregate survey responses [13]. Authors J.C. and K.F. independently grouped survey responses by topic and subsequently generated codes based on these groupings, reviewing the data multiple times in an iterative process in order to devise a codebook that encompassed every response. Individual responses were included in multiple groupings, if appropriate.

J.C. and K.F. subsequently compared and consolidated their initial codes by combining codes that overlapped and discussing the significance of discrepant codes. After settling upon a shared set of codes, they grouped the codes into overarching themes and worked to review, define, and name these themes. Although no formal consensus coding was conducted, author B.D. additionally reviewed the data, codes, and themes.

Descriptive statistical analysis was also performed to assess response rate and basic demographic statistics. No inferential tests were performed.

Reflexivity

The researchers are an internal medicine-pediatrics resident (J.C.), a pediatrics resident (K.F.), and a faculty member in Internal Medicine, Pediatrics, and Divinity, and Director of the Internal Medicine-Pediatrics Residency Program (B.D.) at Yale School of Medicine. J.C. and K.F. are members of the Yale Resident Fellow Senate Wellness Council and were prompted to think about resident perceptions of wellness programming after several interventions carried out by the wellness council received informal negative feedback. They approached this project with curiosity about how to improve GME-sponsored wellness initiatives, and with a willingness to fundamentally change the structure and function of their existing wellness council. B.D. has extensive experience in qualitative research, including on physician thriving.

## Results

Response rate and demographics

The survey was distributed to 1,379 house staff, including approximately 1,025 eligible resident physicians (vs. fellow physicians). Of 162 respondents, 78 were deemed eligible for the study based on the exclusion questions and responded to at least one qualitative question. Of note, comment counts in tables refer to individual coded comments - a single respondent could have comments that applied to multiple codes and/or multiple comments applying to a single code.

These 78 respondents represented at least 18 different residency programs (13 respondents identified as “other” or did not respond), an overall response rate of approximately 7.6% (78/1,025). Internal medicine had the highest representation, with 20 respondents (25.6%), followed by pediatrics (eight respondents, 10.3%), internal medicine-pediatrics (seven respondents, 9.0%), and anesthesiology and general surgery (five respondents each, 6.4%) (Table 1).

**Table 1:** Participant Demographics

Demographic		n (%), N = 78
Gender Identity		
	Female	40 (51.3%)
	Male	25 (32.1%)
	Prefer not to answer/No response	13 (16.7%)
Age		
	26-30	44 (56.4%)
	31-35	21 (26.9%)
	36-40	1 (1.3%)
	No response	12 (15.4%)
Race		
	Asian participants	13 (16.7%)
	Black or African American participants	6 (7.7%)
	White participants	36 (46.2%)
	Other participants	7 (9.0%)
	Prefer not to answer/No response	16 (20.5%)
Do you identify as Hispanic?		
	No	60 (76.9%)
	Yes	1 (1.3%)
	Prefer not to answer/No response	17 (21.8%)
Residency Program		
	Clinical Pathology	1 (1.3%)
	Clinical/Anatomic Pathology	2 (2.6%)
	Diagnostic Radiology	3 (3.8%)
	Emergency Medicine	4 (5.1%)
	Internal Medicine - Pediatrics	7 (9%)
	Internal Medicine - Primary Care	6 (7.7%)
	Internal Medicine - Traditional	14 (17.9%)
	Neurology	1 (1.3%)
	Pediatrics	8 (10.3%)
	Psychiatry	2 (2.6%)
	Therapeutic Radiology	1 (1.3%)
	Anesthesiology	5 (6.4%)
	General Surgery	5 (6.4%)
	Obstetrics-Gynecology	2 (2.6%)
	Ophthalmology	1 (1.3%)
	Orthopedic Surgery	1 (1.3%)
	Otolaryngology	1 (1.3%)
	Cardiothoracic Surgery	1 (1.3%)
	Other/No response	13 (16.7%)

Slight majorities of respondents were between 26 and 30 years old (44 respondents, 56.4%) and identified as female (40 respondents, 51.3%). The largest proportion of respondents identified as White (36 respondents, 46.2%), followed by “prefer not to answer/no response” (16 respondents, 20.5%) and Asian (13 respondents, 16.7%). Six respondents identified as Black or African American (7.7%), and in a separate question, one respondent identified as Latino (Table 1).

Three overarching themes emerged through qualitative analysis - the importance of social connections and personal resources, the impact of clinical and academic work environments, and resident views of administrator-driven programming.

Social connection and personal resources

Respondents generally indicated a desire for social connections and personal wellness resources (Table 2).

**Table 2:** Social Connections and Personal Resources Descriptions of codes under the theme of “Social connections and personal resources,” along with the approximate number of comments related to each code, and sample quotes. Brackets indicate text taken from the survey questions, words that have been edited for clarity, or words edited out to conceal the institution's identity.

Code Name	Description	Sample Quotes
Social connections	Residents want more opportunities for social connection with colleagues, friends, and family	“[I would like to see] more social events between different residency programs…”
(40 comments)		“Time spent with my fellow interns/ friends so we can share our experiences…[seems to positively impact my well-being as a resident]”
		"[I would like to see] more protected time for important life events (family b-day parties…etc.).”
Food	Residents want improved access to nutritious, plentiful food - either for free or at subsidized prices	“I wish there was more food in the resident lounge.”
(64 comments)		"More food is always nice.”
		“[I would like to see] more money for food in the cafeteria…”
		“[I would like to see] better food options for residents at lunchtime.”
Financial concerns/Benefits	Residents want improved financial compensation and benefits	Pay has been the same for several years. Need a raise. [city of institution] is expensive.”
(29 comments)		"[I would like to see] more moonlighting opportunities
		"Paying for parking…[seems to negatively impact my well-being as a resident].”
		"Working to figure out childcare while a resident [seems to negatively impact my well-being]…there need to be better options of residents.”
Fitness	Residents want more resources for pursuing personal fitness	“…Having discounts to local fitness studios…is also very helpful.”
(22 comments)		"…fitness giveaways are always appreciated.”
		"Vouchers for expensive workout classes or free gym memberships…may be more useful.”
Healthcare services	A few respondents note a need for improved access to mental and physical health care	"No time for schedule[d] appointments (mental health, dental, vision, medical) [seems to negatively impact my well-being as a resident].”
(10 comments)		"[I would like to see] expanded mental health therapy sessions beyond the current limit.”
Giveaways/Raffles	Although some respondents appreciate giveaways and raffles, most want different items or view these interventions negatively	"I really enjoyed the free giveaways like socks and bento boxes - that was something to look forward to.”
(31 comments)		"…I would like to see more environmentally conscious sustainable products…”
		"Stop giving us plastic trinkets and corporate gift cards. Invest in local business.”

Many respondents reported positive views of social events and wanted more opportunities to connect socially with colleagues, friends, and family. Beyond the provision of social activities, several respondents indicated a need for more time to facilitate actual participation in social activities or personal life events.

Many respondents also expressed a desire for free or subsidized high-quality food, both at and separate from social events. Some specifically reflected on the lack of convenient, varied, and nutritious food options at work, as well as having limited time to prepare food. Several noted that free food at resident events often runs out.

“Unfortunately, there was not enough food ordered for the resident/fellow lunch. I went to pick up food 15 min after the lunch started and there wasn't any food left. It looked like only 3 platters of sandwiches had been ordered for all of the residents/fellows in the hospital.”

Some respondents reported a desire for improved financial compensation. Others reflected specifically on a need for more benefits, such as free parking, more moonlighting opportunities, and childcare resources. To a lesser extent, respondents indicated a desire for physical fitness resources and for better mental and physical healthcare.

Although some respondents indicated appreciation of giveaways and raffles, most wanted different items or viewed these interventions negatively.

“I want a survey of what we would want for wellness week. Raffles are nice but sometimes we don’t want the items being given and would be happier with a bigger bonus or other items.”

Impact of clinical and academic work environments

Respondents frequently mentioned how aspects of their clinical and academic work environments impact their well-being (Table 3).

**Table 3:** Impact of Clinical and Academic Work Environments Descriptions of codes under the theme of “Impact of clinical and academic work environments,” along with the approximate number of comments related to each code, and additional sample quotes. Brackets indicate text taken from the survey questions, words that have been edited for clarity, or words edited out to conceal the institution's identity.

Code Name	Description	Sample Quotes
Work hours/Time off	Residents want more reasonable work hours and more time off to attend to personal well-being needs	“I would like to see a more robust and straightforward coverage system when people are sick.”
(103 comments)		"[I would like to see]…more efforts towards better hours, more weekends off…”
		"12-15 hour shifts are draining no matter how much you love this job.”
		"[Having] time for my personal relationships and addressing health goals…[seems to positively impact my well-being as a resident].”
Clinical challenges	Challenges at work, including administrative tasks, mistreatment from staff or patients, and inadequate staffing, may negatively impact resident well-being	"[I would like to see more] support [for]…more time with patients (less paperwork).”
(34 comments)		"Being disrespected by care management and nursing [and] doing secretarial work [seem to negatively impact my well-being as a resident].”
		"Being on teams that are inadequate in level of expertise or time available in order to meet patient needs [seems to negatively impact my well-being as a resident].”
Sleep	Some respondents specifically note that the lack of sleep negatively impacts their well-being	"…being tired all the time from lack of sleep and poor sleep…[negatively impacts my well-being as a resident].”
(17 comments)		"Busy 24 hour call shifts without being able to sleep or a break…[negatively impact my well-being as a resident].”
Physical environment	A few respondents note a need for improved physical work environments	"[I would like to see] decent chairs…as well as computers with normal screens (no purple hues) and keyboards that don’t cause carpal tunnel.”
(8 comments)		"Not seeing daylight [seems to negatively impact my well-being as a resident]”
Academic culture	Residents want greater autonomy over and support for professional development, more constructive feedback, and greater respect/ recognition for their work	"Pressure to pursue research or other…extra-curriculars without additional compensation and/or more time off to dedicate to those responsibilities [seems to negatively impact my well-being as a resident.”
(26 comments)		"Working with attendings who are interested in teaching, who are positive and supportive [seems to positively impact my well-being as a resident].”
		"Positive affirmations from leadership…[seem to positively impact my well-being as a resident]”

Regarding clinical work environments, many respondents cited inadequate staffing, exposure to mistreatment or microaggressions from other staff members, and excessive administrative tasks as negatively impacting their well-being.

“Mistreatment from nurses, case managers/ social workers, patients, families, residents, fellows and attendings without reliable report systems, constant micro-aggressions, and lack of required bystander training by faculty [all seem to negatively impact my well-being as a resident].”

To a lesser extent, respondents noted the impact of poorly designed physical workspaces (e.g., access to windows or ergonomic chairs).

Work hours and time off were mentioned most frequently of any topic. Respondents indicated a desire for more time off, more consistent schedules, and time to attend to personal well-being needs.

“[I would like to see more] days off in which I’m able to pursue hobbies, spend time with family, and focus on other things outside of medicine; [I would also like to see] shorter shifts-12-15 hours are draining no matter how much you love this job. My dream is that one day residency will be closer to a regular job…”

Many respondents also specifically mentioned that lack of sleep negatively impacts their well-being.

Regarding academic work environments, many respondents indicated a desire for greater autonomy over and support for personalized professional development.

“[I would like to see] less pressure to do research since not all residents want to pursue this path and considering how many hours we work it seems unfair for that to be a hidden expectation.”

Views on administrator-driven or limited programming

Many respondents expressed views on administrator-driven wellness initiatives and interventions with limited impact (Table 4).

**Table 4:** Views on Administrator-Driven or Limited Programming Description of codes under the theme of “Views on limited or administrator-driven programming,” along with the approximate number of comments related to each code, and additional sample quotes. Brackets indicate text taken from the survey questions, words that have been edited for clarity, or words edited out to conceal the institution's identity.

Code Name	Description	Sample Quotes
Disconnect between GME and residents	Wellness initiatives that do not acknowledge underlying structural determinants of well-being may be seen as disingenuous	“I appreciate the efforts but still feel that amidst …residency programs that do not allow residents to pursue work-life balance that these initiatives can be perceived poorly…”
(26 comments)		"I think it is patronizing to give out ‘Be Well’ stickers.”
		"Having to do modules that emphasize taking care of yourself but not having the ability to take part in activities that do [seems to negatively impact my well-being as a resident]”
Equity	Residents notice when wellness initiatives favor certain groups of residents. These scenarios may negatively impact well-being.	"…it seemed many of the nonsurgical programs had more flexibility and thus opportunities to participate in these events…”
(29 comments)		"Promotion was good. Just not enough for residents who don’t get breaks at noon.”
		"Giveaways being first come first served and items running out [seemed to negatively impact my well-being as a resident]…each resident should have equal access to items.”
Supply	Residents notice when wellness interventions are not provided in sufficient quantities. These scenarios may negatively impact well-being.	"It was a nice gesture but there was too little of everything which further highlighted the things that make residency suck which is lack of control...”
(26 comments)		"Just don’t run out of food when you advertise food.”
		"Would rather not have the…event that ran out of food extremely quickly as it was a bummer coming to empty trays.”
Access	Residents want more interventions that are not limited by time/ date or location. Knowing about interventions but not being able to access them may negatively impact well-being.	"…I was unable to attend due to clinical duties. That’s why the free fitness classes were so nice - we can go on our own time!”
(58 comments)		"[I would like to see] less time restriction…more benefits made available via e-mail.”
		"Having inequality…based on which campus your currently rotating at is unfair.”
		"I was working in the MICU that week…we kept getting e-mails about chair massages and food…which honestly hurt more than helped because we couldn’t get any of it.”

Embedded within comments about work hours and time off, and negative views on certain interventions, respondents expressed a sense that wellness initiatives fail to acknowledge underlying determinants of well-being. Respondents suggested that this sense of disconnect between administrator-driven programming and resident experience is particularly pronounced when wellness initiatives seem to favor certain groups of residents.

“The lunches were not distributed evenly, especially since the [specific program] residents utilize the resident lounge daily and ate all of the food, thus [when] the other residents came down, there was no more food available.”

As this quote also exemplifies, many respondents noted issues with an inadequate supply of wellness interventions, with some indicating that knowing about, but not benefitting from, wellness interventions may negatively impact resident well-being.

Regarding how to improve equity in wellness initiatives, some respondents indicated a specific desire for interventions that are not limited by time or location, thus providing more flexibility for residents completing different clinical rotations, or in multiple programs, to participate.

“[I would like to see] less time restriction for wellness events. More benefits made available via email like [a] free movie ticket or gift card.”

Summary model

**Figure 1:**
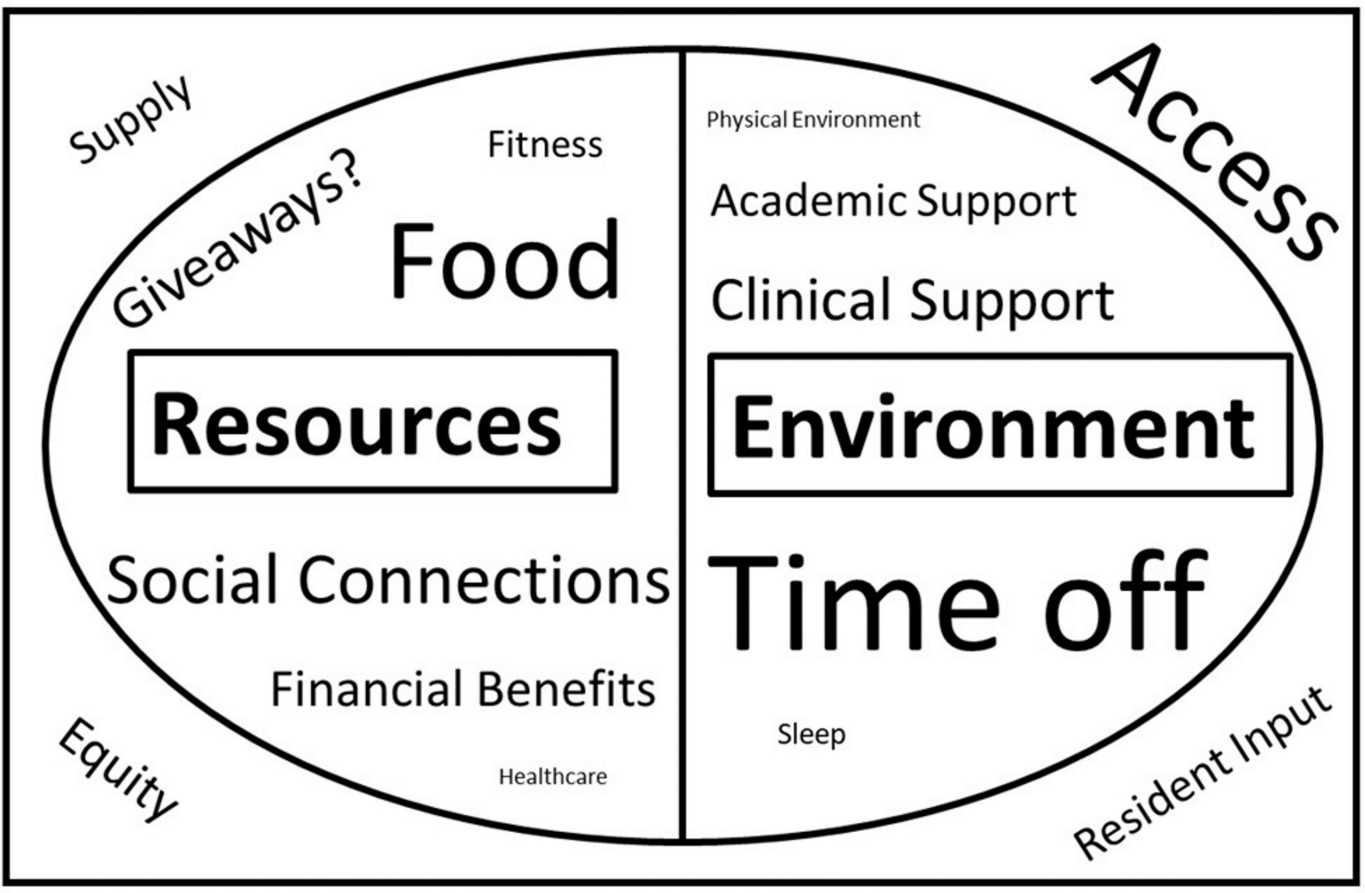
Model for Impactful Wellness Initiatives Proposed model of considerations for designing impactful graduate medical education (GME)-sponsored wellness initiatives for resident physicians. Areas for intervention are divided into resources (i.e., personal resources available to residents) and environment (i.e., ways in which the residency experience can be modified to support well-being). Additionally, initiatives should consider issues of supply, access, and equity between residency programs, and ideally integrate resident input in their design. Relative font sizes reflect the approximate number of comments in this study related to each item.

The themes and underlying codes contribute to a proposed hypothesis-generating model for designing impactful GME-sponsored wellness initiatives for resident physicians. This model divides the area for intervention into resources (i.e., personal resources available to residents) and environment (i.e., ways in which the residency experience can be modified to support well-being). Additionally, initiatives must consider issues such as supply, access, and equity, which may impact residents’ experience of wellness initiatives. This model is summarized in Figure 1.

## Discussion

Our study is unique in that it assessed perceptions of wellness interventions among resident physicians from multiple programs, ultimately highlighting how features of resident work environments - including work hours, time off, and learning climates - impact well-being. This domain is of foundational importance. Although personal resources may play a role in supporting resident well-being, our results suggest that giveaways, raffles, or even free food cannot fully ameliorate a toxic work environment. In fact, many residents noted that superficial projects can paradoxically backfire: morale may decrease when residents recognize inequity in wellness initiatives; “Resident Appreciation Days” or “Wellness Weeks” may function as a disingenuous-seeming panacea in the face of larger structural issues impacting resident well-being.

Our results also echo prior research on the benefits of social connection and support. Social connection is increasingly recognized as having positive physical and mental health benefits, and loss of social connection may contribute to burnout in the healthcare setting [14,15]. Fostering social connection among healthcare professionals has not been well studied, but focused projects show promise for positively impacting work environments [15].

Alongside these conclusions, our themes correspond with existing theoretical models on physician thriving and well-being. For example, our findings closely align with a proposed model for resident well-being based on a modern version of Maslow’s hierarchy of needs, in which the “hierarchies” exist on equal ground, with each area supporting the others [16]. Many of the specific resident needs identified by this model - including food, fitness, sleep, financial security, mental healthcare, and support for social events and family relationships - are re-demonstrated in our themes [16]. This model additionally touches upon the importance of fairness, respect, and access to resources for professional development - needs also echoed by our findings [16]. Another model for physician well-being, based on the “Blue Zones of Happiness” - principles for happy living extrapolated from the study of the world’s “happiest” countries - also encapsulates many of our themes within the categories of physical health, financial stability, social connectedness, purpose in life, and community support [17].

Beyond theoretical models, our findings also complement other qualitative research on physician thriving. Multiple studies identify social connectedness, community, and collegiality as determinants of physician thriving, well-being, and work- and life satisfaction [18-21]. Fairness, respect, and autonomy or agency in the work environment are other recurrent themes [18,19]. Similar to our findings, prior research has also identified work quantity and financial compensation as mediators of work satisfaction [18]. A recent study showing improved burnout, professional fulfillment, and work engagement related to a physician coaching program also corresponds with our respondents’ expressed desire for more constructive feedback and greater support for personalized professional development [22]. Finally, in looking at specific interventions, a 2020 systematic review identified several trends in successful resident wellness initiatives that correspond with our findings, including mentorship, community building, and responsiveness to resident feedback [9].

Limitations and future directions

Notable limitations of our study include uneven representation across residency programs and demographic groups, and a relatively small sample size. It is possible that residents who did not respond to the survey have different perceptions of, and preferences for, wellness interventions that would have shaped our findings. However, because this was an exploratory, qualitative study to identify themes in residents’ perceptions of wellness interventions, we believe that the respondents adequately captured relevant issues and that we ultimately achieved a sample adequate for exploratory qualitative analysis. This was demonstrated by the emergence of thematic saturation in qualitative analysis.

Additionally, to our knowledge, this study was one of the first to explore perceptions of wellness interventions among residents from multiple programs at the same institution. Regardless, studies should seek to further corroborate our findings across other institutions and with larger sample sizes. Future investigations may also assess the impact of specific interventions, in particular, those that enhance learning climates and foster social connection. For example, small studies have shown that regular social dinners with gratitude exercises can improve resident well-being [23].

This project was also a qualitative study employing a survey format, which allowed for anonymity but limited further exploration of expressed themes. Future investigations may further explore these themes through in-depth interviews or assess the impact of specific interventions. Given the dynamic setting in which our research was conducted - with interventions being adapted shortly after preliminary results were collected - there may also be opportunities to assess pre- and post-intervention markers of well-being and perceptions of wellness initiatives.

## Conclusions

Our findings suggest the existence of consistent determinants of physician well-being across specialties, clinical settings, and training levels. These determinants, however, likely encompass a broad range of possible wellness interventions in the GME setting. While personal resources and social events may play a role in supporting resident well-being, these interventions can be perceived poorly if not coupled with efforts to modify work environments toward better supporting resident well-being and personal and professional growth. Furthermore, despite notable consistency in preferred interventions, improving well-being is not a one-size-fits-all task, and wellness initiatives that assume the same preferences and availability for all residents may have the opposite of their desired effects - a finding that further highlights the importance of diversifying GME wellness programming.

## Appendices

Appendix A

Screening for Eligible Participants

Are you a member of the YNHH Wellness Council or were you otherwise involved in planning/implementing activities related to the 2024 YNHH GME Resident/Fellow Appreciation Week?

- Yes --> Excluded from further questions

Are you a clinical fellow who has already completed a separate residency training program? (Residents in integrated residency programs, such as the Solnit Child Psychiatry Program or Integrated Cardiothoracic Surgery, may participate in this survey)

- Yes --> Excluded from further questions

Are you a member of a Yale-affiliated residency program that is not primarily located at the YNHH York-Street or St. Raphael’s Campuses or the West Haven VA Hospital? (e.g., Griffin Hospital Internal Medicine Residency Program)

- Yes --> Excluded from further questions

Demographics

What is your residency program?

- Select residency program

What is your age?

- Enter age

What is your gender identity?

- Male

- Female

- Transgender/male

- Transgender/female

- Other-Write-in

- Prefer not to answer

With which race do you identify most closely?

- American Indian or Alaskan Native

- Asian

- Native Hawaiian or Pacific Islander

- Black or African American

- White/Caucasian

- Other-Write-in

- Prefer not to answer

Do you identify as Hispanic?

- Yes

- No

- Prefer not to answer

Survey Questions

1) How could Yale GME (through the YRFS Wellness Council or otherwise) do a better job of promoting resident well-being? (Free text response)

- What would you like to see more of? (Give examples)

- What would you like to see less of or changed? (Give examples)

2) How could your program do a better job of promoting resident well-being? (Free text response)

- What would you like to see more of? (Give examples)

- What would you like to see less of or changed? (Give examples)

3) Are there wellness initiatives through Yale GME (through the YRFS Wellness Council or otherwise) or your program that you might consider inappropriate, offensive, or otherwise detrimental to your well-being? (Free text response)

4) What factors in particular seem to positively impact your well-being as a resident? (Free text response)

5) What factors in particular seem to negatively impact your well-being as a resident? (Free text response)
